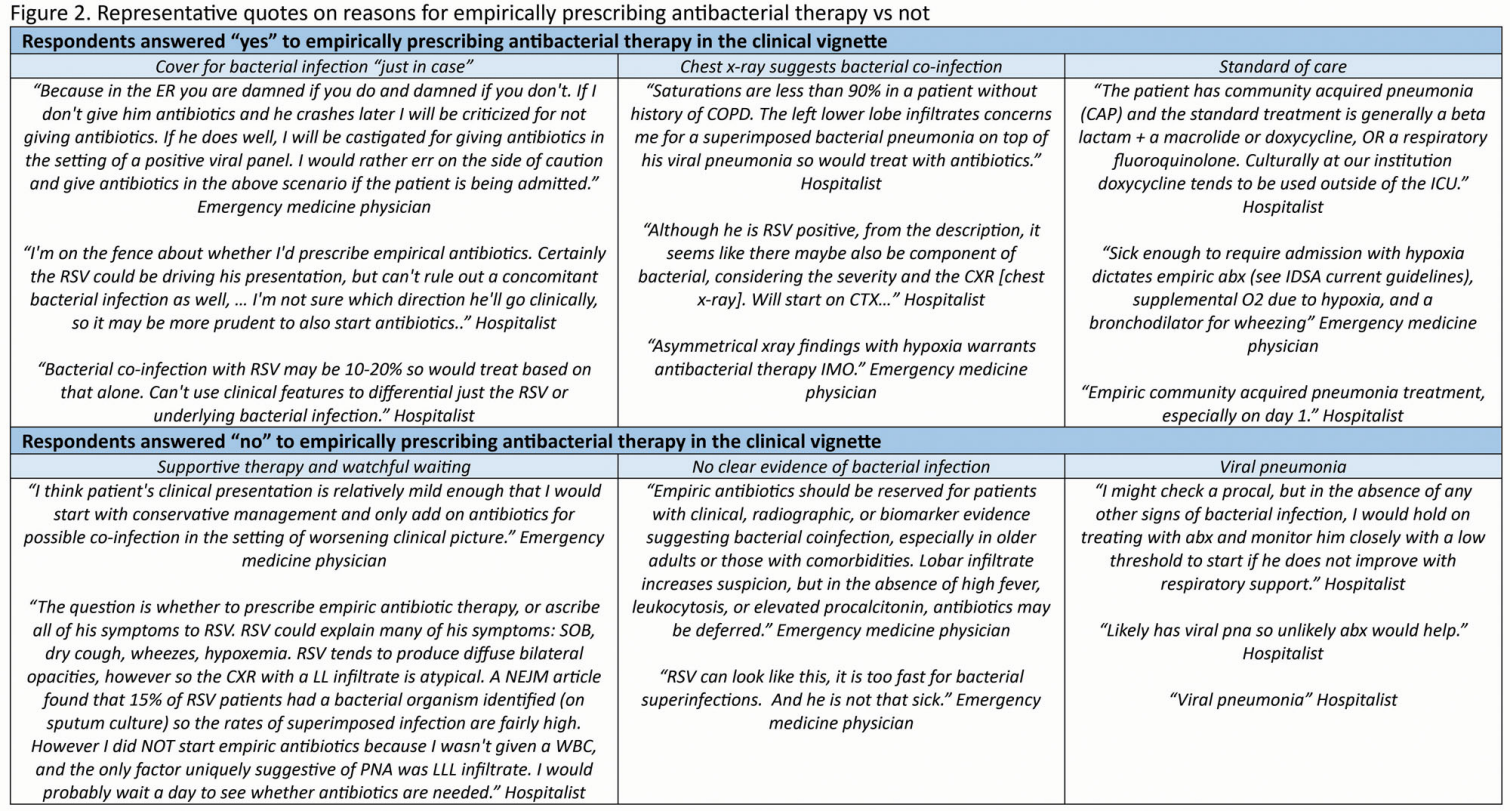# 103 Evaluation of a Preoperative Decolonization Strategy to Prevent Surgical Site Infection in Spinal Fusion Surgery

**DOI:** 10.1017/ash.2026.10521

**Published:** 2026-06-23

**Authors:** Andrea White, Michael Pulia, Hannah Imlay, Theresa Cheng, Allison Bond, Shilpa Raju, James Harrison, Valerie Vaughn

**Affiliations:** 1 University of Utah School of Medicine; 2 University of Wisconsin-Madison; 3 University of Utah; 4 UCSF; 5 Division of Hospital Medicine, University of California San Francisco

## Abstract

**Background:** Community-acquired pneumonia (CAP) is among the top 10 reasons people seek emergency care and the most common indication for antibacterial use overall. Up to 40% of CAP is caused by viruses, now more easily detectable by rapid diagnostics. Although viral CAP is common, evidence on how to best to treat it is lacking—especially with regard to empiric antibacterials. The 2025 American Thoracic Society guidelines recommend viral CAP be treated with empiric antibiotics. In response, the Infectious Diseases Society of America removed its endorsement of the guideline. Here, we used a vignette survey to assess clinical practices and reasoning regarding early treatment of viral CAP. **Methods:** Between 10/2025 and 12/2025, we distributed an online clinical vignette survey to attending hospitalists and emergency medicine (EM) physicians from 3 academic medical centers. The vignette described a patient presenting to the emergency department with signs and symptoms of pneumonia (lobar infiltrate, fever, hypoxia) and a positive test for respiratory syncytial virus (RSV). In the survey, clinicians were asked which treatments they would prescribe, their rationale for doing so, and whether clinicians would be willing to enroll a similar patient in a clinical trial examining the need for empiric antibacterial therapy. **Results:** Respondents included 37 hospitalists and 39 EM physicians (25% response rate, summary in Figure 1). Treatments most commonly selected were supplemental oxygen (93%), inhaled bronchodilators (78%), and empiric antibiotics (63%). Most hospitalists and EM physicians (57% and 69%) selected empiric antibacterial therapy and estimated 72% of their colleagues would also do so. Primary reasons for antibacterial therapy (Figure 2) included covering for a potential bacterial co-infection, evidence suggesting bacterial infection, and local practices. Conversely, physicians who did not select antibacterial therapy favored supportive therapy and observation, interpreted available data as not supporting bacterial infection, and cited local practices. Most (97%) physicians supported enrolling similar patients in a clinical trial examining the need for empiric antibacterial therapy, citing an evidence gap supporting either decision. **Conclusions:** This vignette survey of EM and hospital medicine physicians showed wide variation in hypothetical use of empiric antibacterials for viral CAP. Factors related to treatment with empiric antibacterial therapy included: 1) covering bacterial infection “just in case” vs. watchful waiting, 2) heterogeneous interpretation of incomplete information as supporting vs. not supporting bacterial infection, and 3) local practice patterns. Most physicians (97%) supported conducting a clinical trial to provide evidence to guide empiric antibacterial therapy in viral CAP.

**Figure:**
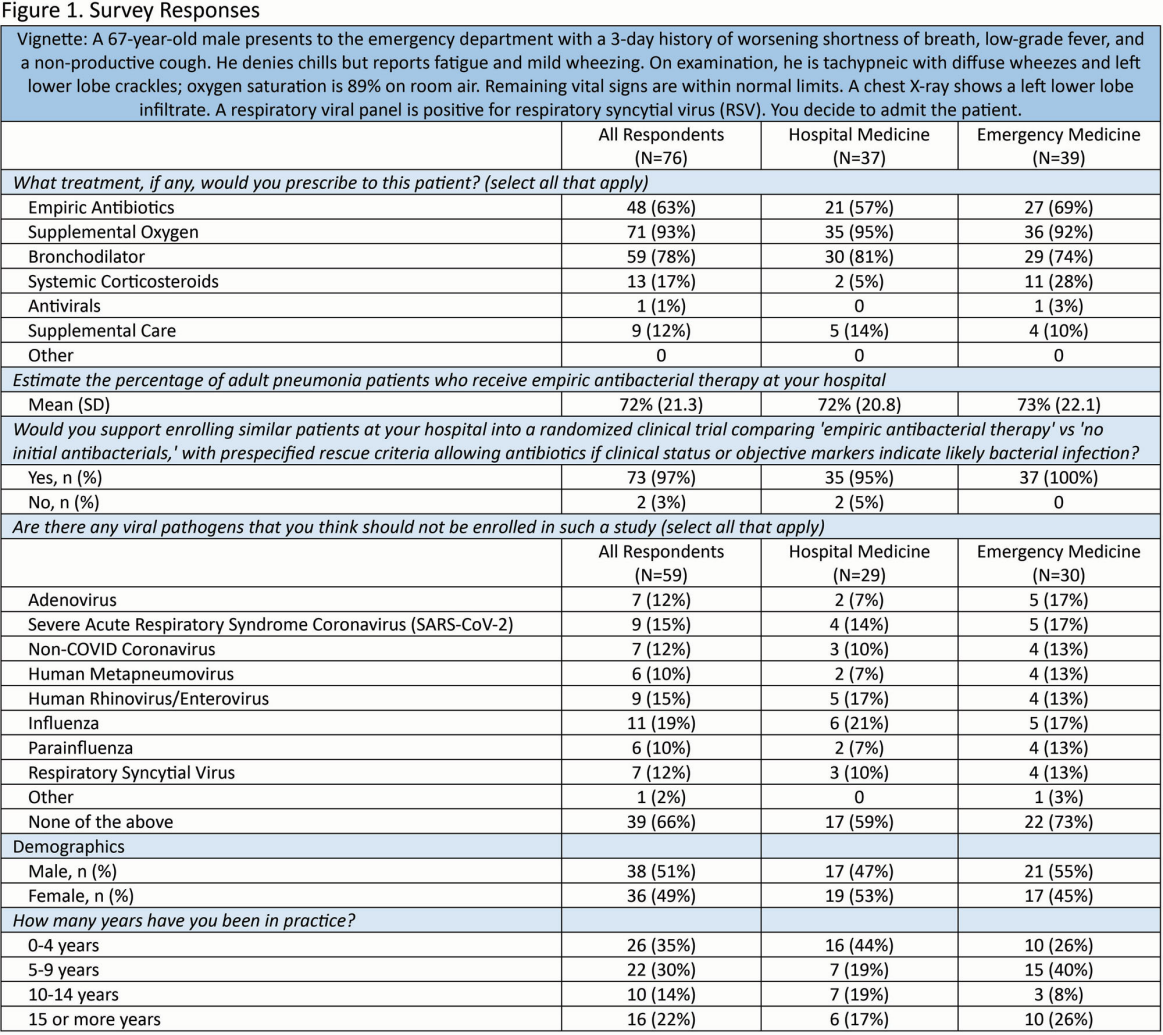

**Figure:**